# The effect of *Cuminum cyminum* on the return of bowel motility after abdominal surgery: a triple-blind randomized clinical trial

**DOI:** 10.1186/s12906-024-04530-1

**Published:** 2024-07-04

**Authors:** Esmaeili Abdar Amin, Elahabadi Ismail, Raeiszadeh Mahboobeh, Sadeghi Tabandeh

**Affiliations:** 1 Medical Surgical Nursing Student, Student Research Committee, School of Nursing and Midwifery, Rafsanjan University of Medical Sciences, Rafsanjan, Iran; 2 Department of Surgery, Ali Ibn Abi Talib Hospital, School of Medicine, Rafsanjan University of Medical Sciences, Rafsanjan, Iran; 3 Herbal and Traditional Medicines Research Center, Kerman University of Medical Sciences, Kerman, Iran; 4 Department of Pediatric Nursing, School of Nursing and Midwifery, Non-Communicable Diseases Research Center, Rafsanjan University of Medical Sciences, Rafsanjan, Iran

**Keywords:** Surgery, Cuminum cyminum, Defecation, Flatulence, Ileus, Complementary therapies

## Abstract

**Background and objectives:**

Considering the significant prevalence of ileus after abdominal surgery and the beneficial effects of *Cuminum cyminum* in digestive problems, this study aimed to examine whether *Cuminum cyminum* has any effect on the return of bowel motility after abdominal surgery.

**Materials and methods:**

In this triple-blind clinical trial study, 74 patients undergoing abdominal surgery were assigned to the intervention and control groups using minimization methods. The patients in the intervention group consumed 250 mg capsules containing *Cuminum cyminum* extract 4 h after the surgery and another dose of the drug 1 h afterward. The patients in the control group consumed a 250 mg capsule containing starch as a placebo at hours similar to those in the intervention group. The instruments used to collect the data were a demographic questionnaire and a researcher-made checklist to assess bowel habits. The data were analyzed using SPSS-22 software.

**Results:**

The average time of gas passing in the intervention and control groups was 9.03 ± 3.41 and 11.72 ± 4.21 h, respectively. The defecation times in the intervention and control groups were 16.97 ± 5.02 and 26 ± 9.87 h, showing a significant difference between the two groups as indicated by the independent samples T-test (*P* > 0.001). Furthermore, abdominal pain, abdominal bloating, nausea, and vomiting were significantly less frequent in the intervention group compared to the control group as confirmed by Fisher’s exact test (*P* > 0.001).

**Conclusion:**

According to the results, the consumption of *Cuminum cyminum* after abdominal surgery helps to reduce the time of gas passing, defecation, and the return of bowel motility. However, additional studies need to address the effectiveness of *Cuminum cyminum* by changing the time and duration of its use.

## Introduction

After the majority of surgeries, especially abdominal surgery, the movements of the gastrointestinal tract are disturbed and it takes time for the gastrointestinal tract to return to its normal function [1]. This complication most commonly occurs after intraperitoneal surgery and the more extensive the surgery, the longer the recovery time [2]. Although the mechanism underlying this disorder is unknown, the dysfunction of the parasympathetic system, which leads to intestinal nerve reflex disorder and the loss of peristalsis, the activation of inhibitory neuron pathways, and the release of inflammatory factors are among the potential causes proposed for this disorder [3].

Bowel motility disorder after abdominal surgeries is the most common cause of delayed discharge of patients from the hospital [4]. The delay in the return of bowel motility, which is seen in almost half of the patients after surgery, is a nervous, hormonal, or medicinal process, and has various consequences such as abdominal distention, decreased bowel sounds, delayed gas passing and defecation, increased pain after surgery, delay in wound healing, nausea and vomiting, loss of appetite, and delay in starting oral feeding [4, 5]. In addition to affecting the patient after surgery, this disorder also significantly increases the cost of healthcare systems [3]. Usually, three factors, the time of the first gas passing, the time of the defecation, and the time of hearing the first bowel sound are considered symptoms of the return of bowel motility, and the change in the time of the return of bowel motility affects these three factors [6].

There are various methods to improve bowel motility after surgery, but eating orally is recommended as the most basic method to reduce the duration of bowel obstruction. Indeed, when the food enters the gastrointestinal tract, the intestinal reflexes are stimulated and improve the motility and stimulation of intestinal hormones [1]. Despite various studies around the world, there is still not enough medical evidence on how to improve the return of bowel motility after surgery [3]. In a review article, Lubbers et al., investigated the role of the vasovagal system in ileus after surgery and concluded that the use of anti-inflammatory drugs reduces the incidence of ileus symptoms [7]. Furthermore, Rahmani et al., concluded that intraperitoneal bupivacaine causes the return of bowel motility faster after surgery [8]. Chen et al., examined the effect of percutaneous electrical acupuncture on the return of bowel motility after abdominal surgery and showed that this intervention can be an effective and safe treatment in this field [5]. Huang et al., also concluded that the use of intravenous Dexmedetomidine accelerates the return of bowel motility after laparoscopic nephrectomy surgery [9]. Various studies have also confirmed the effect of chewing gum in improving the return of bowel motility [1, 4, 10–13]. However, in textbooks, there is no standard treatment for postoperative ileus, and currently, surgeons do not know any other standard method to suggest to patients other than reassurance about the return of bowel motility [3].

Due to the side effects of chemical drugs in recent decades, there has been a growing desire to use herbal drugs, and complementary medicine treatments have been increasingly used in many countries as low-risk, affordable, and easy treatment options with limited side effects [14]. For example, Khadem et al., concluded that topical chamomile oil has a potential therapeutic effect on gastrointestinal motility and can reduce the duration of post operative ileus [15]. One of the herbal plants introduced in traditional medicine to improve the digestive condition is *Cuminum cyminum*, which has anti-bloating, pain-relieving, intestinal antiseptic, and appetizing effects, and can contribute to emptying the stomach and bowels [16].

*Cuminum cyminum* is a small herbaceous plant with delicate and aromatic leaves, belonging to the family of sedges, 15–30 cm in length, whose origin was the banks of the Nile River in Egypt, but today it is growing in large areas of the Mediterranean, Arabia, and Iran [17]. This plant has tannin, resin oil, and essential oil [18]. It inhibits the cholinergic receptors of the smooth muscles of the digestive tract due to its antispasmodic and anti-flatulent effects [18]. So far, no side effects or toxic effects have been reported from the consumption of this plant and it is safe for human consumption [19]. The mechanism of action of *Cuminum cyminum* in the treatment of flatulence, indigestion, and other digestive complications has been attributed to strengthening the peristalsis bowels and helping to empty the stomach and intestines [16].

In this context, Sakhavar and Mirteimoori showed that *Cuminum cyminum* is similar and even more effective than Magnesium hydroxide in reducing gastrointestinal complications after emergency cesarean Sect. [20]. Fazel and Esmaili also confirmed the effectiveness of cumin oil in reducing the severity of bloating after cesarean surgery [21]. Moreover, Rajabi et al., concluded that cumin has an effect equivalent to mefenamic acid in the treatment of painful abdominal spasms [22]. Babaei et al., also showed that *Cuminum cyminum* extract is effective in reducing the residual volume of the stomach in traumatic patients under ventilators in the intensive care unit [16]. Singh et al., also reported that the consumption of *Cuminum cyminum* relieves indigestion symptoms such as bloating, abdominal distension, and pain [23]. Al-Snafi, also suggested that *Cuminum cyminum* extract can protect the mucous layer of the digestive tract by increasing and regenerating mucin [24]. However, to the best of the researchers’ knowledge, no study has yet addressed the effect of *Cuminum cyminum* on the return of bowel motility after abdominal surgery. Therefore, considering the long history of Persian medicine, and the emphasis of the World Health Organization on using the experiences of different human races with traditional medicine [3, 25], this study aimed to examine the effect of *Cuminum cyminum* on the return of bowel motility after abdominal surgery.

## Materials and methods

### Study design

The participants in this triple-blind randomized clinical trial (IRCT20150713023190N10, 24/10/2021) were patients undergoing abdominal surgery in the surgery department of Ali Ibn Abitaleb hospital of Rafsanjan, southeast of Iran from November 2021- May 2022.

### Sampling and randomization

Following a similar study [6], the sample size was calculated using the two-mean-comparison formula

$${n_1} = \frac{{{{\left( {{Z_{1 - \frac{\alpha }{2}}} + {Z_{1 - \beta }}} \right)}^2} \times \left( {\sigma _1^2 + \frac{{\sigma _2^2}}{k}} \right)}}{{{\Delta ^2}}},\,{n_2} = k \times {n_1}$$

with an effect size of (Δ = 4) for gas passing as well as σ1 = 3.73, σ2 = 5.83, K = 1, α = 0.05 and β = 0.1. (The significance level was set at *p* < 0.05, and the study power was assumed to be 90%). In addition, a sample size of *n* = 31.40 was determined for this study. Given the possibility of sample dropout, 37 patients were allocated to each group.

The inclusion criteria in the study were as follows: Age between 18 and 60 years, candidate for abdominal surgery (open appendectomy, laparoscopic cholecystectomy, and laparotomy), having general anesthesia, not performing laparotomy due to gastric perforation, cooperation and consent to participate in the study, sign the written informed consent form, not using *Cuminum cyminum* regularly, no pregnancy and lactation, no drug addiction, no use of alcohol, no concurrent diseases (history of liver disorders, kidney failure, heart failure, and thyroid disorders), no use of anticoagulants, and no use of tranquilizer and sedative drugs. The exclusion criteria were as follows: failure to take one or both doses of the drug or placebo, discharge of the patient earlier than the scheduled time (24 h), exacerbation of the patient’s condition, occurrence of allergic symptoms or reactions after taking the first dose of drug or placebo, and death of the patient.

The participants were randomly assigned to two groups using the minimization method. To do so, the patients were first categorized based on key variables, such as type of surgery and gender. Afterward, the first participant was placed in the intervention or control group by coin flipping, and other participants with lower scores on the research variables were allocated to the study groups. In the case of equality, random allocation was repeated.

### Instruments

The data were collected using a demographic checklist for the patient (age, gender, education, marital status, type of surgery, and receiving analgesia during the intervention) and a checklist for assessing the return of bowel motility (time of the first gas passing, time of the first defecation, frequency of bloating, abdominal pain, nausea, and vomiting). This checklist prepared based on the related textbook and past article [26]. Both of times in the checklist recorded by hour and other variables recoded each two hours until 24 h as yes and no. For gaining more accurate information, a table was developed based on the checklist to be completed by the patients or their caregivers and was handed to the researcher after the 24 h. Validity of the instruments was assessed and confirmed using the content validity based on experts’ opinions.

### Drug preparation

The capsules used in this study were prepared by the Research Center for Herbal and Traditional Medicines, Kerman University of Medical Sciences, Kerman, Iran. The dry fruits of *Cuminum cyminum* were purchased from one of the herbal medicine stores in Kerman, Iran. For extraction, coarse powder of dry fruit was soaked in ethanol 70% for 48 h and then filtered through Whatman filter paper using a Buchner funnel under vacuumed pressure. The extraction process was repeated three times, and finally, the hydroalcoholic extract of *Cuminum cyminum* was concentrated in a rotary evaporator and desiccated in an oven at 45 °C. The dried hydroalcoholic total extract was packed in 250 mg capsules.

In order to standardize *Cuminum cyminum* extract, the amount of total phenolic and flavonoid contents was measured using folin–Ciocalteau and aluminum chloride colorimetric method, respectively.

The Folin-Ciocalteu method was used for total phenolic content measurement based on a recent report [27]. In brief, standard solutions of gallic acid were prepared in ethanol (12.5–200 µg/mL). 0.1 mL of *Cuminum cyminum* extract or gallic acid solution was mixed with 0.5 mL of Folin-Ciocalteu reagent. Subsequently, 0.4 mL of sodium carbonate (7.5%) was added and after a 30 min ancubation at room temperature and darkness, the absorbance was measured at 765 nm by utilizing a multi-mode microplate reader (BioTek®, USA). The total phenolic content was calculated from the calibration curve of gallic acid (Y = 0.0052x + 0.103, R^2^ = 0.995). The outcome results were expressed as mg of gallic acid per gram of dried extract.

The aluminum chloride colorimetric method was used for the determination of total flavonoid content of *Cuminum cyminum* extract based on quercetin standard [28]. One mL of quercetin solution in 80% methanol (25–200 µg/mL) was diluted with one mL of distilled water and 100 µL of 5% nitrite sodium was added to the dilution and was shaked slightly for 6 min. Afterwards, 200 µL of 10% aluminum chloride solution was added to the mixture and incubated for 5 min. Finally, one mL of NaOH (1 N) was added to the mixture and the absorption was spectrophotometrically measured at 510 nm aiming to a standard curve of quercetin (Y = 0.005x-0.049, R^2^ = 0.991). The same steps were repeated by *Cuminum cyminum* extract solution. The data were the average of triplicate experiments expressed as mg quercetin/g total extract.122.2. The level of total phenolic and flavonoid content of *Cuminum cyminum* total extract was found to be 104.80 ± 4.25 mg galic acid/g extract and 122.23 ± 5.11 mg quercetin/g extract, respectively.

### Intervention

Each patient in the intervention group received a capsule containing 250 mg of *Cuminum cyminum* extract 4 h after the surgery. The researcher made sure that the patients were fully awake as confirmed by the surgeon. Vital signs also monitored before prescribing the medicine to ensure the stability of the blood pressure after the operation. One hour later, another dose of the drug was given to the patients to ensure the effective concentration in one day [6, 21]. The safety of the drug was confirmed by Sahoo et al. [29]. In Iran, Babaei et al. found no toxic complications for a dose of 3 g per day of *Cuminum cyminum* [16]. The patients in the control group received a 250 mg capsule containing corn starch as a placebo at hours like those in the intervention group. Then, the checklist for evaluating the return of bowel motility was completed by the patients.

**Fig. 1:**
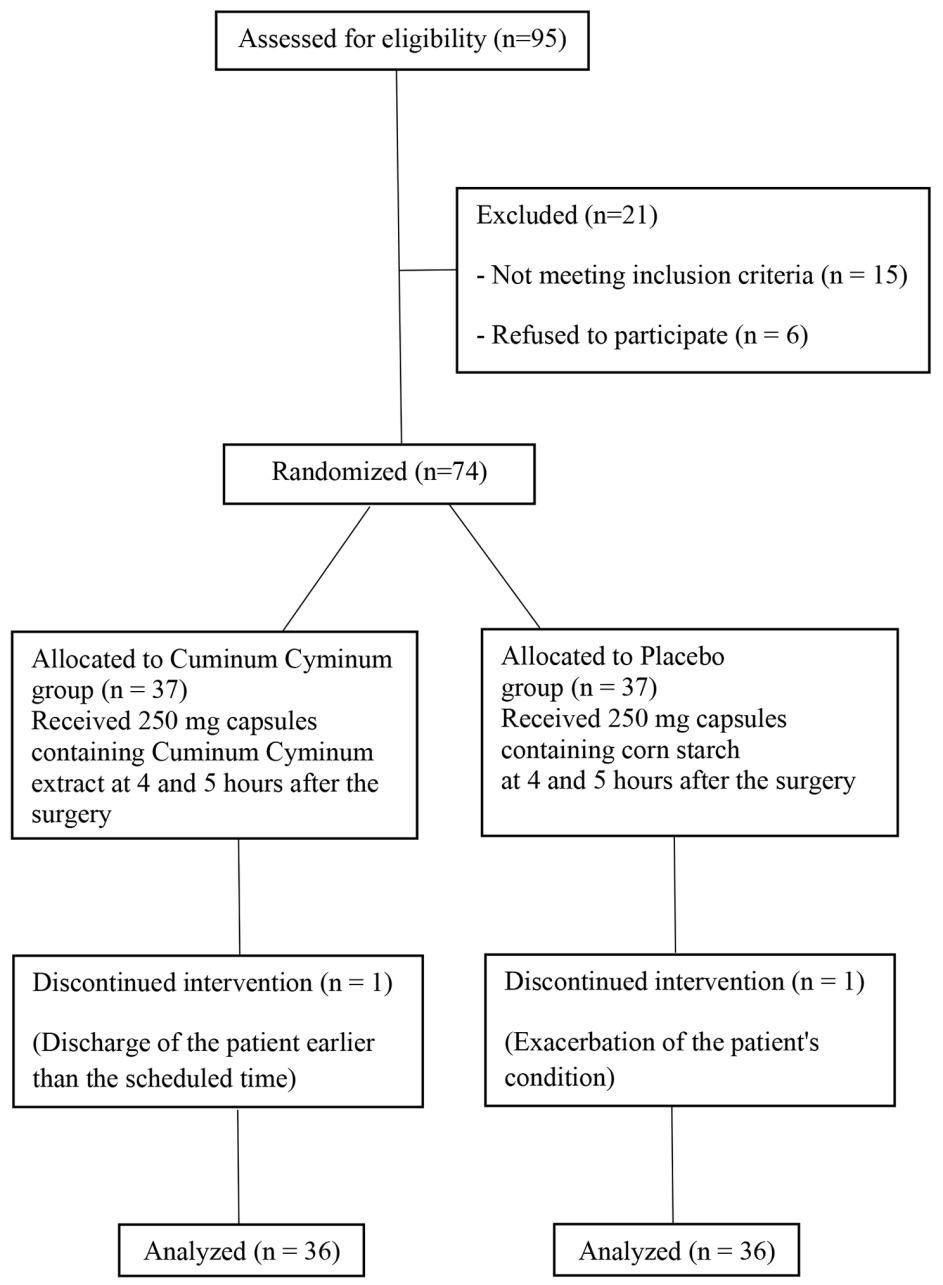
CONSORT chart

After the start of the intervention and under the supervision of the researcher, the patients and their caregivers in both groups were asked to record the time of the first gas passing and the first defecation until the moment of discharge from the hospital. Moreover, bloating, abdominal pain, nausea, and vomiting were recorded in the checklist every 2 h until 24 h after the operation. The researcher, the patient, and the analyzer were blinded to whether the capsules were drug or placebo, and the pharmacist was asked after the analysis. Finally, 1 person in the *Cuminum cyminum* group was excluded from the study due to the earlier discharge than the scheduled time, and 1 person in the placebo group was excluded from the study due to the worsening of the patient’s condition (Fig. 1).

### Analysis

The data were analyzed using descriptive statistics, such as mean and standard deviation, as well as analytical statistics, including the independent samples t-test and the Fisher’s exact test with SPSS Software V.22.

## Results

The patients in both groups were homogenous in terms of demographic characteristics (Table 1). The Kolmogorov–Smirnov test was used to assess the normal distribution of the data, and since the quantitative variables had a normal distribution (p˃0.05), parametric tests were used to analyze the quantitative data.

**Table 1:** Comparison demographic characteristics of patients between two groups

Demographic data		Intervention group Number (%)	Control group Number (%)	*P*-value
Gender	Male	18 (50)	18 (50)	p*=1
	Female	18 (50)	18 (50)	
Type of surgery	Open appendectomy	12 (33/3)	12 (33/3)	P*=1
	Laparoscopic cholecystectomy	12 (33/3)	12 (33/3)	
	Laparotomy	12 (33/3)	12 (33/3)	
Received of analgesia	Apotel	30 (83/3)	30 (83/3)	P*=1
	Narcotics	6 (16/7)	6 (16/7)	
Educational level	Under diploma	11 (30/06)	11 (30/06)	P*=0/95
	Diploma	18 (50)	17 (47/2)	
	Above diploma	7 (19/4)	8 (22/2)	
Marital status	Married	12 (33/3)	13 (36/1)	P*=0/8
	Single	24 (66/7)	23 (63/9)	
Age (year)	(mean ± SD)	33/81 ± 10/54	34/91 ± 12//09	P**=0/67

* Chi-square test

** Independent T-test

The average time of gas passing in the intervention and control groups was 9.03 ± 3.41 and 11.72 ± 4.21 h, respectively. The defecation times in the intervention and control groups were 16.97 ± 5.02 and 26 ± 9.87 h, showing a significant difference between the two groups as indicated by the independent samples t-test (*p* < 0.001) (Table 2). Fisher’s exact test showed that the frequency of abdominal pain in the patients in the intervention and control groups was 12 vs. 21 (*p* = 0.03) 6 to 8 h after surgery and 13 vs. 22 (*p* = 0.03) 14 to 16 h after surgery. Moreover, the two intervention and control groups showed a significant difference in abdominal pain 16 to 18 h after surgery (4 vs. 16, *p* < 0.01) but they did not have any significant difference in other times.

**Table 2:** Comparison the mean and standard deviation of the time of gas passing and defecation between two groups

Variable	Group	(Mean ± SD)	Df	T	*P-value
Gas passing (hour)	*C. Cyminum*	9/03 ± 3/41	70	-2/98	0/004
	Placebo	11/72 ± 4/21			
Defecation (hour)	*C. Cyminum*	16/97 ± 5/02	70	-4/89	< 0/001
	Placebo	26 ± 9/87			

* Independent T-test

The frequency of bloating in the patients in the intervention and control groups was 16 vs. 28 (*p* < 0.01) 8 to 10 h after surgery, 9 vs. 24 (*p* < 0.01) 10 to 12 h after surgery, and 4 vs. 13 (*p* < 0.02) 12 to 14 h after surgery, showing significant intergroup differences, but there was no significant difference between the two groups in terms of frequency of bloating in other times. Moreover, the frequency of nausea in the patients in two intervention and control groups was 2 vs. 10 (*p* < 0.02) 10 to 12 h after surgery, 3 vs. 12 (*p* < 0.02) 12 to 14 h after surgery, 1 vs. 8 (*p* < 0.03) 16–18 h, 0 (*p* = 0) 20–22 h, and 0 (*p* = 0) 22 to 24 h after surgery, showing significant intergroup differences, but there was no significant difference between the two groups in terms of frequency of nausea in other times. Finally, the frequency of vomiting in the patients in the intervention and control groups was 4 vs. 12 (*p* < 0.04) 0 to 2 h after surgery, zero (*p* = 0) 12 to 14 h after surgery, zero (*p* = 0) 20–22 h after surgery, and zero (*p* = 0) 22 to 24 h after surgery, showing significant intergroup differences, but there was no significant difference between the two groups in terms of frequency of vomiting in other times.

## Discussion

The data in the present study revealed a significant statistical difference between the two groups of patients undergoing abdominal surgery in terms of the time of gas passing and defecation after the intervention. The results indicated that the consumption of *Cuminum cyminum* decreased the time of gas passing and defecation in patients in the intervention group. The pathogenesis of ileus has not been completely known, and it seems that it is a multifactorial problem. Based on some studies, ileus could be due to an inflammatory process related to surgical manipulation of the intestine during surgery that leads to significant leukocyte infiltration of muscular layer of intestine [15]. In the Persian medicine, *Cuminum cyminum* is considered stimulant, anti-inflammatory, carminative and astringent and its therapeutic effects have been described on gastrointestinal disorders [23]. A review of the literature found no similar study. However, other studies have addressed other interventions. For example, Cha et al., examined the effect of chewing gum on postoperative ileus in elderly patients after hip fracture surgery. Unlike the results of the present study, the findings showed no significant difference between the first time of gas passing and defecation after hip fracture surgery [13]. These conflicting findings can be attributed to the differences in the type of intervention, research population, and research settings. Mohsenzadeh Ledari et al., examined the effect of gum chewing after cesarean section on the return of bowel function. Although the type of intervention, the research population, and the research setting were also different from the present study, the findings confirmed that chewing gum reduces the time of gas passing and defecation [30]. Moreover, Safdari et al., compared the effects of gum chewing with early initiation of oral feeding and routine regimen on the recovery of bowel function in primiparous women after cesarean Sect. [31]. The results indicated that the average time of gas passing and the time of the first defecation in the intervention group were lower than in the control group.

Abdullahi et al., examined the effect of chewing gum on bowel movements after appendectomy and found that the time of gas passing and defecation in the intervention group was less than in the control group [32]. In addition, Safdari et al., examined the effect of initiation time of oral hydration on the return of bowel function and woman’s satisfaction after elective cesarean section in primiparous women, and the results showed that the first time of gas passing and defecation showed significant differences between the two groups, and these times were less in the intervention group [33]. Yousefi et al., investigated the effects of Carum Carvi (Bunium persicum Boiss) on the early return of bowel motility after the cesarean section and the findings indicated that the average time of gas passing and defecation was lower in the intervention group as was confirmed in the present study [6]. Likewise, Huang et al., examined the impact of intravenous Dexmedetomidine on postoperative bowel movement recovery after laparoscopic nephrectomy and found that the first time of gas passing and defecation were less in the intervention group [9]. In this study, chemical medicine was used to accelerate the return of bowel motility, but herbal medicine was used in the present study, which is less complicated and less expensive than chemical medicine. Traditional uses of *Cuminum cyminum* include reducing inflammation, increasing urination, preventing gas and suppressing muscle spasms [23]. Furthermore, it is more available and acceptable to patients [9].

The data in the present study showed that abdominal pain, bloating, nausea, and vomiting were less frequent in the patients in the intervention group. In line with the present study, Rahmani et al., examined the effects of intra-peritoneal Bupivacaine on post-operative bowel motility and pain and reported that abdominal pain, nausea, and vomiting were significantly less frequent in the intervention group than in the control group [8]. However, the two studies used different methods. Furthermore, Schuster et al., examined the effect of chewing gum after open sigmoid colectomy on the ileus and found that nausea and abdominal pain were less frequent in the intervention group than in the control group [34]. Using *Cuminum cyminum* as a medicinal plant is more affordable and easily available to the patient. *Cuminum cyminum* inhibits the cholinergic receptors of the smooth muscles of the digestive tract due to its antispasmodic and anti-flatulent ingredients such as tannin, resin oil and essential oils [18]. Vador et al., also showed that the methanolic extract of *Cuminum cyminum* reduced blood glucose and wound score in diabetic rats in comparison with the control group. Moreover, *Cuminum cyminum* significantly increased gastric mucus content, antioxidant status, and cellular adenosine triphosphatase enzyme (ATPase5) level compared to the control group. Methanolic extract of *Cuminum cyminum* prevented the formation of glycosylated end products in vitro and in vivo [35]. The findings of the present study revealed that *Cuminum cyminum* reduced abdominal pain, nausea, and vomiting in patients undergoing abdominal surgery compared to the control group. The mechanism of *Cuminum cyminum* in the treatment of flatulence, indigestion, and other digestive complications has been attributed to strengthening the peristalsis bowels and helping to empty the stomach and intestines [16]. The antispasmodic effect of *Cuminum cyminum* is related to the inhibitory effects on smooth muscle contractions induced by the spasmogens, acetylcholine and histamine [36]. Furthermore, Sahoo et al., showed that *Cuminum cyminum* significantly inhibited the frequency of diarrhea and intestinal fluid secretion compared to the control [29]. The present study showed that *Cuminum cyminum* is effective in increasing bowel motility. In accordance with this study, two review studies [23, 36] reported cumin may be effective in changing intestinal movements by affecting the anesthetized nervous system of the digestive system during surgery. These effects can support the beneficial effects of *Cuminum cyminum* on the return of bowel motility after abdominal surgery based on its pathophysiological nature, i.e., inflammation and neural process.

No study in the literature has reported the negative effect or ineffectiveness of *Cuminum cyminum* on the return of bowel motility. A significant finding in the present study was the increase in bowel motility in the intervention group after consuming *Cuminum cyminum*. Not including the patients’ history of prior constipation or bloating as a potential confounding factor were one of the limitations of the present study. Another limitation of the present study was the inclusion of only patients who underwent abdominal surgery. Thus, future studies should focus on patients undergoing other surgeries taking varying doses of *Cuminum cyminum*.

## Conclusion

*Cuminum cyminum* can be used as an herbal medicine to reduce the time of gas passing and defecation and return of bowel motility among patients after surgery. However, further studies are recommended in this field.

## Data Availability

The datasets used and/or analyzed during the current study available from the corresponding author on reasonable request.

## References

[1] Bosc C, Hebbard DP (2021). Dietary interventions for the reduction of postoperative Ileus following abdominal surgery: a literature review: postoperative Ileus Dietary interventions. J Canada’s Physician Assistants.

[2] Domen A, Stabel C, Jawad R, Duchateau N, Fransen E, Vanclooster P (2021). Postoperative ileus after laparoscopic primary and incisional abdominal hernia repair with intraperitoneal mesh (DynaMesh®-IPOM versus Parietex™ Composite): a single institution experience. Langenbeck’s Archives Surg.

[3] Ghorat F, Khadem E, Shirazi M, Rahimi R, Bioos S, Khademi A (2015). Physiopathology and treatment of post-operative Ileus in viewpoint of traditional medicine and classic medicine: a review article. Med Hist.

[4] Abouda Abdelhamed A, Sarhan Eldesokey A, Nabil Malk R, Elzeblawy Hassan H (2018). Chewing gum: post-operative effect on women’s recovery and bowel motility following gynecologic abdominal surgery. Egypt J Health Care.

[5] Chen KB, Huang Y, Jin XL, Chen GF (2019). Electroacupuncture or transcutaneous electroacupuncture for postoperative ileus after abdominal surgery: a systematic review and meta-analysis. Int J Surg.

[6] Yousefi S, Sadegh Pour O, Hamzeh Gardeshi Z, Sohrabvand F (2019). The effects of Carum Carvi (Bunium Persicum Boiss) on early return of bowel motility after caesarean section: Double-Blind, randomized, placebo-controlled trial. J Family Reproductive Health.

[7] Lubbers T, Buurman W, Luyer M (2010). Controlling postoperative ileus by vagal activation. World J Gastroenterol.

[8] Rahmani Boeni N, abrahimi M, Khalilian AR, Alvandipour M, Sayadi S (2012). The effects of Intera-Peritoneal Bupivacaine on Post Operative Bowel Motility and Pain. J Mazandaran Univ Med Sci.

[9] Huang SS, Song F, Hu S, Yang S, Wang S, Zhao L, Wu Q (2021). Impact of Intravenous Dexmedetomidine on postoperative Bowel Movement Recovery after laparoscopic nephrectomy: a Consort-Prospective, randomized, controlled trial. World J Clin Cases.

[10] Atkinson C, Penfold CM, Ness AR, Longman RJ, Thomas SJ, Hollingworth W (2016). Randomized clinical trial of postoperative chewing gum versus standard care after colorectal resection. Br J Surg.

[11] de Leede EM, van Leersum NJ, Kroon HM, van Weel V, van der Sijp JRM, Bonsing BA (2018). Multicentre randomized clinical trial of the effect of chewing gum after abdominal surgery. Br J Surg.

[12] Liu Q, Jiang H, Xu D, Jin J (2017). Effect of gum chewing on ameliorating ileus following colorectal surgery: a meta-analysis of 18 randomized controlled trials. Int J Surg.

[13] Cha Y-H, Nam DC, Song S-Y, Yoo J-I (2021). A prospective randomized controlled trial to evaluate effect of chewing gum on postoperative ileus in elderly patient after hip fracture. Med (Baltim).

[14] Zaghari Tafreshi M, Rasouli M, Tabatabaee A, Golmakani E, Mortazavi H (2014). Utilization of complementary and alternative medicine in nursing with emphasis on the touch therapy. J North Khorasan Univ Med Sci.

[15] Khadem E, Shirazi M, Janani L, Rahimi R, Amiri P, Ghorat F (2018). Effect of topical chamomile oil on postoperative bowel activity after cesarean section: a randomized controlled trial. J Res Pharm Pract.

[16] Babaei-Abandansari S, Bagheri-Nesami M, Gholipour-Baradari A, Yazdani-cherati J, Yosefi S (2019). The prophylactic effect of *Cuminum Cyminum* Extract on gastric residual volume in traumatic patients under Ventilator hospitalized in Intensive Care Unit. J Med Plants.

[17] Allaq A, Sidik N, Abdul-Aziz A, Ahmed I (2020). Cumin (*Cuminum Cyminum* L.): a review of its ethnopharmacology, phytochemistry. Biomedical Res Therapy.

[18] Steinegger E, Hänsel R. Lehrbuch Der Pharmakognosie: auf phytochemischer Grundlage. Springer-; 2013.

[19] Tafazoli M, Khadem Ahmadabadi M, Asili J, Esmaili H (2013). Comparison the effects of Cuminum and Mefenamic Acid on after pains in Multiparous Women. Iran J Obstet Gynecol Infertility.

[20] Sakhavar N, Mirteimoori M (2009). Comparison of *Cuminum Cyminum* with milk of magnesia in prevention of gastrointestinal discomforts after. J Babol Univ Med Sci.

[21] Fazel N, Esmaili H (2005). The effect of cumin oil on the flatulence intensity after cesarean section. Feyz J Kashan Univ Med Sci.

[22] Rajabi Naeeni M, Modarres M, Amiin G, Bahrani N (2013). A comparative study of the effects of Cumin and Mefenamic Acid capsules on secondary Dysmenorrhea due to IUD: a Randomized Triple Blind Clinical Trial. Complement Med J.

[23] Singh RP, Gangadharappa H, Mruthunjaya K. Cuminum cyminum–A popular spice: an updated review. Pharmacognosy J.2017;9(3).

[24] Al-Snafi A (2016). The pharmacological activities of *Cuminum Cyminum* -A review. IOSR J Pharm.

[25] Zhang X, Organization WH. Traditional medicine strategy 2002–2005. 2002.

[26] Hatefi A, Sadeghi T, Emtiazy M (2018). Comparing the effect of golghand and psyllium on constipation among the elderly: a randomized clinical trial. J Med Plants.

[27] Mehrabani M, Amirkhosravi A, Farhadi S, Vasei S, Raeiszadeh M, Mehrabani M, editors. The influence of harvest time on total phenolic and flavonoid contents, antioxidant, antibacterial and cytotoxicity of Rheum Khorasanicum root extract. Annales Pharmaceutiques Françaises; 2023. 10.1016/j.pharma.2022.11.010 36402205

[28] Amini F, Amini-Khoei H, Haratizadeh S, Setayesh M, Basiri M, Raeiszadeh M (2023). Hydroalcoholic extract of Passiflora incarnata improves the autistic-like behavior and neuronal damage in a valproic acid-induced rat model of autism. J Traditional Complement Med.

[29] Sahoo HB, Sahoo SK, Sarangi SP, Sagar R, Kori ML (2014). Anti-diarrhoeal investigation from aqueous extract of *Cuminum Cyminum* Linn. Seed in albino rats. Pharmacognosy Res.

[30] Mohsenzadeh Ledari F, Barat S, Nasiri Amiri F, Aghajani Delavar M, Banihosseini S, Khafri S (2012). Effect of gum chewing after cesarean-delivery on return of bowel function. J Babol Univ Med Sci.

[31] Safdari-Dehcheshmehi F, Salehian T, Parvin N, Akbari N (2011). Comparison of the effects of gum chewing with those of early initiation of oral feeding and routine regimen on recovery of bowel function in primiparous women after cesarean section. SJKU.

[32] Abdollahi AA, yazdi K, Behnampur N, Neyaze M (2011). The effect of chewing gum on bowel movements after appendectomy. J Arak Uni Med Sci.

[33] Safdari Dehcheshmeh F, Salehian T, Safari M, Akbari N, Deris F, Noorbakhshian M (2012). Effect of initiation time of oral hydration on the return of bowel function and woman’s satisfaction after elective caesarean section in primiparous women. J Gorgan Univ Med Sci.

[34] Schuster R, Grewal N, Greaney GC, Waxman K (2006). Gum chewing reduces Ileus after Elective Open Sigmoid Colectomy. Arch Surg.

[35] Vador N, Jagtap AG, Damle A (2012). Vulnerability of gastric mucosa in Diabetic rats, its pathogenesis and amelioration by *Cuminum Cyminum*. Indian J Pharm Sci.

[36] Johri R (2011). Cuminum cyminum and Carum carvi: an update. Pharmacogn Rev.

